# Training in the implementation of sex and gender research policies: an evaluation of publicly available online courses

**DOI:** 10.1186/s13293-024-00610-6

**Published:** 2024-04-03

**Authors:** Annika Gompers, Madeline T. Olivier, Donna L. Maney

**Affiliations:** 1 Rollins School of Public Health, Emory University, Atlanta, GA USA; 2 Department of Psychology, Emory University, Atlanta, GA USA; 3 Harvard-Radcliffe Institute, Cambridge, MA USA

**Keywords:** SABV, SGBA, Sex differences, Gender differences

## Abstract

**Background:**

Recently implemented research policies requiring the inclusion of females and males have created an urgent need for effective training in how to account for sex, and in some cases gender, in biomedical studies.

**Methods:**

Here, we evaluated three sets of publicly available online training materials on this topic: (1) *Integrating Sex & Gender in Health Research* from the Canadian Institutes of Health Research (CIHR); (2) *Sex as a Biological Variable: A Primer* from the United States National Institutes of Health (NIH); and (3) *The Sex and Gender Dimension in Biomedical Research*, developed as part of “Leading Innovative measures to reach gender Balance in Research Activities” (LIBRA) from the European Commission. We reviewed each course with respect to their coverage of (1) What is required by the policy; (2) Rationale for the policy; (3) Handling of the concepts “sex” and “gender;” (4) Research design and analysis; and (5) Interpreting and reporting data.

**Results:**

All three courses discussed the importance of including males and females to better generalize results, discover potential sex differences, and tailor treatments to men and women. The entangled nature of sex and gender, operationalization of sex, and potential downsides of focusing on sex more than other sources of variation were minimally discussed. Notably, all three courses explicitly endorsed invalid analytical approaches that produce bias toward false positive discoveries of difference.

**Conclusions:**

Our analysis suggests a need for revised or new training materials that incorporate four major topics: precise operationalization of sex, potential risks of over-emphasis on sex as a category, recognition of gender and sex as complex and entangled, and rigorous study design and data analysis.

**Supplementary Information:**

The online version contains supplementary material available at 10.1186/s13293-024-00610-6.

## Abstract (plain language summary)

Recently implemented research policies requiring the inclusion of females and males have created an urgent need for effective training in how to account for sex, and in some cases gender, in biomedical studies. We evaluated three publicly available online trainings on this topic: (1) *Integrating Sex & Gender in Health Research* from the Canadian Institutes of Health Research; (2) *Sex as a Biological Variable: A Primer* from the United States National Institutes of Health; and (3) *The Sex and Gender Dimension in Biomedical Research*, developed as part of “Leading Innovative Measures to Reach Gender Balance in Research Activities (LIBRA)” from the European Commission. We reviewed each course with respect to their coverage of (1) What is required by the policy; (2) Rationale for the policy; (3) Handling of the concepts “sex” and “gender;” (4) Research design and analysis; and (5) Interpreting and reporting data. All three discussed the importance of including males and females to better generalize results, discover potential sex differences, and tailor treatments to men and women. The interconnectedness of sex and gender, how to operationalize sex, and potential downsides of focusing on sex more than other sources of variation were minimally discussed. Notably, all three courses explicitly endorsed invalid analytical approaches that lead to incorrect findings of differences. Our analysis suggests a need for revised or new training materials that cover four major topics: precise operationalization of sex, attention to potential risks of over-emphasizing sex, consideration of gender and sex as complex and intertwined, and rigorous study design and data analysis.

**Supplementary Information:**

The online version contains supplementary material available at 10.1186/s13293-024-00610-6.

## Highlights

Three major online trainings on implementing sex and gender research policies from Canada, the United States, and the European Union covered much of the same content.

A common theme among the trainings was the importance of including males and females to better generalize results, discover potential sex differences, and tailor treatments to men and women.

Topics that were not substantially addressed in the trainings included the extent to which sex and gender are inextricably entangled, the operationalization of sex using concrete, measurable variables, and the potential risks of focusing on sex more than other sources of variation.

All three courses explicitly endorsed invalid analytical approaches that produce bias toward false positive discoveries of difference.

Key areas for improved or new materials are: precise operationalization of sex, attention to the downsides of over-emphasis on sex as a category, recognition of gender and sex as complex and entangled entities, and rigorous study design and data analysis.

**Supplementary Information:**

The online version contains supplementary material available at 10.1186/s13293-024-00610-6.

## Background

To redress a longstanding androcentric bias in biomedical research, funding agencies and journals around the world have instituted policies requiring the inclusion of females as well as males in most research studies [1–4]. Such policies have typically mandated consideration of sex (and in some cases, gender) in health research. For example, Canada’s *Health Portfolio Sex- and Gender-Based Analysis* (SGBA) Policy came into effect in 2009. SGBA is “an analytical process” that, according to the Canadian Institutes of Health Research (CIHR), must be used “to develop, implement and evaluate the Health Portfolio’s research, legislation, policies, programs and services to address the different needs of women and men,” including by “integrating sex and gender into health research when appropriate” [2]. Accordingly, in 2010, CIHR began requiring all grant applicants to respond to questions about whether “sex as a biological variable” and “gender as a socio-culture factor” will be considered in the research [5].

Other countries have issued similar requirements. The United States National Institutes of Health (NIH), for example, enacted their policy *Sex as a Biological Variable* (SABV) in 2016. This policy states: “NIH expects that sex as a biological variable will be factored into research designs, analyses, and reporting in vertebrate animal and human studies” [1]. All researchers applying for NIH funding must now “account for sex” in their applications. Proposals that do not describe plans to report data disaggregated by sex or that describe plans to study a single sex, without strong justification, are expected to receive less competitive scores by reviewers [6].

The European Commission has also instituted research policies around sex and gender [3]. As early as 2002, requirements were put into place to address the “gender dimension,” meaning the integration of sex and/or gender into research design. Today, Horizon Europe, the European Union’s main research funding program for 2021–2027, requires all proposals to address sex and gender wherever appropriate [4]. This policy is part of a much larger initiative to address and eliminate “gender inequality and intersecting socio-economic inequalities – including those based on disability, ethnicity, and LGBTIQ – throughout research and innovation systems, including by addressing unconscious bias and systemic structural barriers” [4].

The full intent of these policies, to increase not only inclusion but also rigor and reproducibility, has yet to be realized. Although biomedical research is now more likely to include females and males, few studies include sex as a factor in their experimental designs [7–9]. Further, even when sex is included as a factor, appropriate analytical approaches are infrequently employed [10]. Studies of policy implementation have identified gaps in researcher knowledge of the policies and how to implement them [11, 12], for example whether sample sizes must be doubled and how to properly statistically test for sex differences.

Given that funding agencies in North America and Europe require consideration of sex and gender regardless of a researcher’s training and research interests, educational and training materials for researchers are vital. To this end, several funding entities have developed and/or provided support for online courses intended to provide instruction about the rationale for the policies and how to comply with them. Here, we reviewed the content of three such online courses, one each from Canada, the United States, and the European Union. From Canada, we reviewed CIHR’s *Integrating Sex & Gender in Health Research*. Introduced in 2015, the CIHR materials were shown in a 2018 study to improve knowledge and self-efficacy in the consideration of sex and gender in research as well as to increase motivation to implement the policies [13]. From the United States, we reviewed the *SABV Primer*, which was introduced by the Office for Research on Women’s Health (ORWH) in 2020 and covers how to consider SABV in all stages of the biomedical research spectrum. To our knowledge, the European Union does not offer training in a format similar to the NIH and CHIR offerings; we therefore reviewed materials developed as part of a project funded by Horizon 2020 called “Leading Innovative measures to reach gender Balance in Research Activities” (LIBRA). Active from 2015 to 2019, LIBRA worked to raise awareness of the need for sex and gender to be integrated into all phases of research (referred to as the “sex and gender dimension in research,” or SGR); as part of that effort, online training modules were made available on their website. These materials, called *The Sex and Gender Dimension in Biomedical Research*, comprise a defined set of modules containing learning objectives and quizzes.

Our goal in reviewing each of the three courses was to evaluate the quality and usefulness of the information presented. We reviewed coverage of each of five topics. First, we looked at how the policies themselves were explained and the extent to which the trainings clearly communicated what is being asked of researchers. Second, we noted how the trainings represented the rationales for the policies, for example to improve generalizability of findings or unmask variation. Third, we evaluated how the concepts “sex” and “gender” were handled, attending particularly to the extent to which sex and gender were understood to be inextricably entangled vs. dissociable [14, 15]. We also noted guidance related to how sex or gender categories should be operationalized or contextualized, for example how to choose a concrete, quantifiable variable such as chromosome complement or reproductive anatomy to represent sex category vs. relying solely on undefined categories [16–19]. Fourth, we evaluated recommendations on research design and analysis, particularly when it related to the statistical analysis of subgroup data [20]. Finally, we noted any guidance about interpretation and reporting of results, for example how to represent sex-related findings in scientific publications and communicate those findings to the public.

## Methods

### Data sources

Detailed descriptions of each course we reviewed, including the links to each, are presented in Supplemental File 1. Each of the three courses requires learners to create a free account to access the material, which is self-paced in all cases. *Integrating Sex & Gender in Health Research* was developed by CIHR and will be referred to hereafter as simply “CIHR.” It consists of three separate modules, each with a number of objectives, in slideshow format with text, images, and narration. Each module includes multiple choice questions with immediate feedback and short, open-ended questions throughout. The *SABV Primer*, developed by ORWH at NIH, will be referred to hereafter as “NIH” or “the Primer.” Like the CIHR training, the Primer is formatted as slideshow presentations with text, images, and narration. It is organized into four modules, each with five or six individual lessons. At the end of each module, learners answer several multiple-choice questions with immediate feedback. *The Sex and Gender Dimension in Biomedical Research*, which we will refer to as “LIBRA,” is organized into video lectures, slide presentations, and case studies. Each LIBRA module concludes with a multiple-choice quiz.

Materials were accessed from CIHR between December 28, 2022 and March 28, 2023, NIH between January 13 and February 7, 2023, and LIBRA between May 15 and May 19, 2023 (see the descriptions of the trainings for the URLs). We prepared the materials in each training by converting them into formats convenient for coding. PowerPoint slide presentations were made from CIHR trainings, the NIH Primer, and the portions of the LIBRA training that were conducive to such (Module 2 and the Case Studies in Module 3). These presentations included any spoken narration as transcribed text. The remaining portions of LIBRA (Module 1 and a portion of Module 3), which consisted of oral presentations, were transcribed into Word documents with any relevant visuals embedded in those documents.

### Data analysis

We iteratively developed codes for evaluating the three trainings. One author (AG) reviewed the entire NIH Primer and summarized the major topics and issues. A second author (DLM) organized the themes into 73 discrete codes (Table S1). AG and DLM then independently reviewed all three trainings, coding content in Excel as appropriate. The list of codes was altered only slightly during data collection; a code for “disaggregation of data is beneficial for meta-analysis” was added shortly after coding began. After all training materials were coded by AG and DLM, the two Excel spreadsheets were combined and synthesized into summaries based on overarching themes. Summaries were reviewed by both of these authors and any disagreement or factual inaccuracies were resolved through discussion until a consensus was reached.

As an addendum to our review, one author (MTO) conducted internet searches to identify other relevant trainings on how to implement SABV and similar policies. Our search terms and the dates of these searches are presented in Table S2.

## Results

All three trainings stated learning objectives that included understanding the advantages of considering sex and/or gender, mastery of proper terminology, best practices in research design, and how to evaluate other studies. Below, we summarize our findings regarding the content.

### Rationale for sex and gender research policies

Each of the trainings emphasized the need for and importance of sex and gender research policies, often citing the same rationales. Generalizability was a major focus of each training, with the explicit or implicit notion that results from studies on one sex or studies that do not account for sex cannot be generalized to both females and males. NIH explicitly stated that “the results of a study with subjects of a single sex cannot be generalized to the other” and that not accounting for sex leads to “erroneous conclusions” or “erroneous assumptions that results apply to both sexes.” LIBRA stated that overgeneralization occurs “when the study is conducted in one sex but results are presented as if they apply to both sexes.” CIHR similarly stated that overgeneralization may occur when sex is not accounted for, and that “there is a risk of harm by assuming that the study results apply to everyone.” Risk of harm by not considering sex and gender in research was frequently leveraged in all three trainings to highlight the importance of sex and gender policies. Few examples of harm were offered; in all three courses, they were taken primarily from a 2001 US Government Accountability Office (GAO) report about increased risk of adverse drug events in women. This report itself [21] clarifies that most of the drugs with adverse events mentioned in the report were prescribed mostly to women, which explains the disparity in adverse events [22, 23]; however, none of the trainings mentioned this detail.

A second rationale for sex and gender research policies, referenced in each of the trainings, was reproducibility. The NIH Primer relied most heavily on reproducibility as a motivating factor, and typically paired it with references to rigor and transparency with statements such as: “SABV is a key focus of the NIH initiative to enhance reproducibility in biomedical research through rigor and transparency in studies.” CIHR’s usage of reproducibility was narrower, limited to the issue that there are “problems with reproducibility when the sex of cells, tissues, and animals are not explicitly recorded and reported.” One of the videos in the LIBRA training stated that depositing raw data by sex (or race, as mentioned by the speaker) “is key for reproducibility.”

“Precision medicine” was frequently leveraged as a rationale for sex and gender research policies, with references to the promise of sex-specific treatments appearing in all three trainings. CIHR stated that discovering sex differences will “improve health by tailoring treatments differently for men and women.” NIH stated explicitly that SABV will “allow better translation for personalized sex-specific treatment” and called for not only sex-specific therapeutic interventions but also sex-specific recommendations for clinicians and policymakers. LIBRA was more focused in its invocation of sex-specific medicine, describing the findings of its two case studies as leading to potential diagnostic markers specific to women.

Both CIHR and NIH emphasized that including males and females will further understanding of mechanisms underlying sex differences related to health; CIHR stated, “Sex matters in biomedical research… because mechanisms are needed to explain observational similarities and differences in the epidemiology of the disease under study, as well as response to treatment.” Whereas NIH suggested that investigating underlying mechanisms was not a requirement of SABV policy, CIHR emphasized that research including females and males needs to “include a clear objective to elucidate the mechanisms of any differences/similarities that may arise.” In one of the research scenarios offered by CIHR, for example, principal investigators were criticized for not proposing to show how the outcome measures were affected by hormones, even though exploring such mechanisms was not a primary focus of their study.

Both the CIHR and NIH trainings contained somewhat vague references to efficient use of resources. According to CIHR, “inefficiencies may occur” if sex is not accounted for at all stages of design, analysis, and reporting. NIH warned that not incorporating SABV results in “wasted money and resources” and “failure to maximize return on investment.”

### Descriptions of sex differences

Common examples of sex differences given in the trainings included differences in substance use, cardiovascular disease, pain, and mental health. The nature and size of sex differences were often described using hyperbolic language; for example, the NIH Primer stated that there is “abundant evidence that there are distinct biological differences between females and males,” and repeatedly referred to these as “fundamental” or “basic” differences. LIBRA similarly described sex differences as self-evident, for example that “it’s very well known that there are big differences between males and females” and that “sex hormones are obviously very important translationally and during the clinic.” While CIHR contained comparatively less hyperbolic language, it did include statements such as, “the influence of sex on health extends from the cellular to the societal level” and that “sex should be analyzed at all levels, from chromosomes and cells to whole organisms.” This sentiment was echoed by the Primer: “Sex and gender factors can be addressed distinctly from cells to selves.”

### Sex and gender: definitions and operationalization

All three trainings emphasized the importance of terminology, particularly a distinction between the terms “sex” and “gender.” LIBRA considered the confusion of sex and gender to be one of the three main mistakes in sex and gender research. In all the trainings, sex was equated with “biology.” Gender was defined as “socially constructed” and largely behavioral by CIHR, cultural by NIH, and socio-cultural by LIBRA. Gender was noted in all three trainings to apply to humans only, although CIHR allowed for “rare exceptions, such as in research involving animal behaviours that are dependent on context or environment.” One of the speakers in the LIBRA course referred to the “gender” of mice. Both sex and gender were implicitly or explicitly defined as binary throughout all three trainings, whether through language such as “both sexes,” “both women and men,” “the opposite sex,” or through lack of discussion of intersex or transgender individuals. CIHR occasionally presented an intersex symbol on a slide, and repeatedly referred to “gender-diverse people” as distinct from men, women, boys, and girls, without elaboration. Only CIHR noted that gender can change over time. None of the trainings mentioned that sex can change in some species; CIHR noted specifically that sex cannot change.

All three trainings stated that both sex and gender are relevant to health, but sex was presented as more relevant to biological research. SABV (NIH’s policy) addresses only sex, but SGBA (CIHR’s policy) and SGR (LIBRA’s framework) include both sex and gender. The trainings noted the entanglement of sex and gender to varying degrees. Referring to this entanglement, the Primer used the language “inextricably linked,” CIHR used “interconnected” or “interacting,” and LIBRA used “interact.” CIHR suggested the usage of “sex/gender” when the two are inseparable, and pointed out that some observed sex differences could be explained by gender. However, many differences were asserted to certainly be caused by sex, such as a difference in kidney function.

Operationalization of sex differed among the courses. According to NIH, sex is “encoded in DNA” and defined by chromosomal complement (XX vs. XY). While this chromosomal definition was repeated many times throughout the Primer, other options for operationalization of sex were presented on one of the slides, including self-report or observation. CIHR defined sex as a set of attributes associated with chromosomes, gene expression, and hormone levels. LIBRA did not explicitly define or operationalize sex but did note that one advantage of studying non-human animals is that “the sex variable can be broken down into its constituent parts,” referring to chromosomes and hormones. CIHR and NIH instructed researchers to “properly identify” the sex of animal models, tissues, or cells (CIHR) and report operational definitions (NIH). However, no explanations or examples of proper operationalization or determination of sex were given in any of the trainings.

All three trainings referred to the sex of cultured cells and argued that the chromosomal complement of a cell defines its sex. NIH and LIBRA both asserted that “every cell has a sex.” CIHR stated that “cells and tissues can generally be classified as female or male by the chromosomal complement,” a view reiterated by NIH and LIBRA. Despite blanket references to “male and female cells” and instructions to “take into account the sex of cells,” there were instances in which the trainings acknowledged the complexity and controversy regarding whether and how cells can be “sexed.” The Primer noted, for example, that “NIH recognizes current challenges to the authentication of the sex of established cell lines.” LIBRA mentioned the complicated interplay between chromosomes and androgen receptors in cell culture and further noted that “primary cells may obscure sex differences because of the in vitro environment.”

Other than the idea that gender is distinct from sex, the NIH and LIBRA training materials did not contain further information about its operationalization. Operationalization of gender was, in contrast, a major focus of the CIHR training. Module 3 in particular covered gender scales and methods for measuring gender. The challenges noted included working with secondary datasets that do not contain sufficient information and anticipating all gender-related variables. Ultimately, however, CIHR advised to not adjust for variables such as social support, employment status, and education because these differ for men and women and adjustment would therefore erase the effect of gender.

### Generalization of sex-related findings from non-human animals to humans

In all three trainings, it was assumed or explicitly stated that sex differences in non-human animals can be generalized to humans. We noted one exception during the expert interview in LIBRA, when the interviewee remarked that mouse strain is a key factor in generalizability and replicability of findings: “Another very important thing that is probably not adequately stressed even by funding agencies is that… there are many strains of mice, which have a different susceptibility to cancer. So even there, unless you argue that you want to validate all your experiments in different mouse strains, not only males and females, you can see that it’s actually becoming impossible.” Nonetheless, in one case study in LIBRA, results from a single experiment in one strain of knockout mice were generalized to recommend a strategy for assessing colon cancer risk in women.

### When to include females and males

A major focus of each training was how to decide whether to include females and males or conduct a single-sex study. All the trainings emphasized the importance of literature searches to determine what is already known about sex differences in the area of interest. CIHR and LIBRA placed this material in the context of human prevalence, arguing that including males and females is important when there are known sex differences in the condition being studied or modeled. For example, CIHR noted that a female-only study of the mechanisms underlying bladder cancer in mice was “not scientifically sound” because the prevalence of the disease is four times higher in men than women. Both LIBRA and CIHR recommended including females and males when evidence of sex differences is absent or equivocal.

Advice about inclusion was less straightforward when the literature showed evidence of sex similarities. CIHR presented a scenario in which researchers were using only male mice “to keep the numbers of animals to a minimum.” The learner was instructed that, because the literature showed that the sexes do not differ with respect to the mechanism under study, this reasoning is sound. There was, however, neither an explanation of why a single-sex study is better than one that also includes females, given the established lack of a sex difference, nor a consideration for the potential cost of discarding females. The advice also appeared to conflict with the instruction for human studies that “if data from early phase trials do not indicate potential sex-related differences, it cannot be assumed that clinically relevant differences do not exist.”

All three trainings endorsed a single-sex approach when the condition being studied occurs primarily in one sex, for example prostate cancer (LIBRA) or breast cancer (CIHR). Single-sex approaches were also deemed acceptable when a condition had already been studied in one sex and researchers want to study another (LIBRA). Limiting a study to one sex simply because previous studies were conducted in that sex was deemed unacceptable, however (CIHR).

The trainings differed with respect to whether single-sex studies are justified when resources are limited. NIH condoned single-sex studies when animals are scarce, such as non-human primates. At the same time, NIH also argued that cost is never an acceptable reason to exclude one sex. CIHR recommended that when feasibility is the justification for a single-sex study, that choice must be acknowledged as a limitation and the implications for impact must be considered.

A major point made by both CIHR and LIBRA was that inclusion of females and males is not necessary in studies of “basic” biology. For example, CIHR claimed that sex is not relevant to understand protein-protein interactions and other molecular mechanisms, and inclusion of males and females would “not strengthen the quality” of such studies. Similarly, LIBRA argued that sex is “clearly” not relevant in studies of protein-protein interactions. Notably, none of the trainings offered a rationale or evidence supporting an advantage of single-sex approaches to studying molecular processes. On the contrary, a major overall theme of all three trainings was that basic, protein-based mechanisms, such as drug-receptor interactions and regulation of gene expression, do differ between the sexes. Although CIHR insisted that “there are no sex differences in protein-protein interactions” the next sentence stated that there are “different” pharmacokinetic mechanisms in males and females. For CIHR, the advice to conduct single-sex studies of molecular processes was overridden by a sex difference in prevalence of a related condition; in an example research scenario, researchers were studying an asthma-related protein in adult mice. Because there were no known sex differences in the protein, the researchers proposed a single-sex study. Despite the lack of sex differences at the molecular level, however, CIHR deemed the study not scientifically sound because in human children, asthma is more common in boys than girls.

A substantially different endorsement of single-sex studies was offered by LIBRA. In Module 2, learners were told that “single-sex studies are an obvious choice” for researchers interested in how “cells or animals differ according to age, hormonal status, circadian cycle, etc.” That is, LIBRA seemed to say that sex is not relevant when the independent variable of interest is something *other* than sex. By this logic, longitudinal studies looking at changes over development or changes over the circadian cycle should not be required to include females and males. This advice, given both in the instructional and quiz portions of LIBRA Module 2, presented an interesting contrast with both NIH and CIHR policy.

### Accounting for hormones and hormonal cycles

Advice about tracking ovarian cycles sometimes seemed to conflict, even within a particular training. CIHR noted in Module 1 that “consensus among experts suggests that controlling for fluctuations in gonadal hormones in initial experiments is unnecessary.” Yet, later in the same module, researchers were advised to “acknowledge how variability in endogenous hormone levels will be accounted for.” Elsewhere in Module 1, CIHR advised researchers to consider documenting or controlling hormonal status “where appropriate,” which was clarified as cases in which “there is evidence that reproductive hormone variability affects the dependent measure.” Similarly, NIH’s Module 2 argued that according to meta-analyses, females are not more variable than males when estrous cycles are not controlled; earlier in the same module, however, the training stated that “researchers working with animal models should consider the influence of male and female hormones and the hormonal cycle in experimental design.” LIBRA drew a distinction between cycles in rodents and those in humans; it was argued in Module 1 that the rodent estrous cycle is too short for gene transcription to change from phase to phase (an incorrect assertion) [24, 25]; in contrast, researchers studying premenopausal women were advised to track the stage of cycle.

### Research designs and reporting results

#### Exploratory vs. confirmatory research and statistical power

NIH emphasized that choices about experimental design, particularly relating to power, depend on whether detection of sex differences is a main goal of the study. In this way, NIH distinguished between research intended to confirm sex differences and research that is exploratory in nature: “consider whether your intent is to (1) look for sex differences OR (2) to appropriately consider and control for sex when evaluating the effect of your experimental condition or intervention.” The same directive, although emphasized less, was found in LIBRA: Whether to power your study “depends on whether you’re interested in [sex differences] or not.” CIHR’s position on the matter was more nuanced. In Module 1, learners were instructed to always test for sex differences, even when underpowered: “Large differences can often be detected even with small sample sizes.” For human clinical studies in particular, however, CIHR advised always powering to detect sex differences. On the same slide, it was recommended that assessing sex differences should be planned “once the overall treatment effect has been shown to be significant,” suggesting that if the treatment was not effective when the sexes were considered together, then there would be no reason to test for sex differences. This suggestion seemed to conflict with directives elsewhere in the training, as well as on that slide, that data should always be disaggregated by sex “in order to identify potential differences in dose response.”

Neither CIHR nor LIBRA offered detailed guidance about how to calculate power. LIBRA’s advice was simply to “consult a statistician.” LIBRA went on to advise that a sample size of eight would never offer enough power whereas a sample size of 16 is ideal, but no power calculation or discussion of effect size was presented to support these statements. CIHR offered only that power analyses should always be done. Further, in the CIHR training, nearly every mention of power co-occurred with a mention of including males and females in equal numbers; these two concepts were used interchangeably at times. Only NIH went into more detail about how power is calculated but their guidance did not take sex as a variable into account. In Module 3, NIH advised that if a researcher’s goal is to detect sex differences in response to treatment, a power analysis must be conducted and the study powered accordingly. Subsequently, instructions were provided on how to calculate power for a comparison between treated and untreated groups; sex as a variable was not considered, however, and no guidance was presented on how to calculate power to detect either the effect of interest within each sex or a sex difference in the response to treatment. We noted NIH’s recommendation that “a t-test will yield the most accurate result in your power analysis” (a t-test is, to our knowledge, not a method for calculating power).

The trainings disagreed with each other about whether sample sizes must be increased to consider the influence of sex or gender. CIHR noted that power analyses are likely to show that sample size must be increased. LIBRA reiterated this concern, noting that researchers conducting power analyses are likely to see the required numbers of animals “skyrocket.” LIBRA spent considerable space on the issue, covering the balance between “statistical significance” and the financial and ethical cost of including more animals. LIBRA went so far as to say that sufficient power to compare the sexes can be accomplished only with a doubling of sample size. In contrast, NIH pushed back against the idea that including females and males always requires a doubling of sample size, emphasizing that main effects of treatment can usually be detected in a group of males and females just as easily as a single-sex group without increasing numbers overall. It is important to note, however, that whereas LIBRA’s point was about detecting sex differences in the response to treatment, NIH’s point was about detecting main effects of treatment, not comparisons of those effects between sex. NIH’s insistence that sample size does not need to be increased conflicted with their guidance throughout the rest of the training that data be disaggregated by sex for analysis and reporting, which would profoundly reduce power unless sample size were increased. Perhaps to mitigate the loss of power, NIH stated, “For some designs that consider SABV, but are not intended to detect sex differences, examination of the data will allow the observation of potential trends in the data related to sex. Decisions can then be made whether to follow up with a study explicitly designed and powered to detect sex differences.”

#### Analyzing and reporting sex-based data

A major theme of all three trainings was that data should always be analyzed separately for females and males. LIBRA stated, for example, “Study outcome measures, that is the effects of treatment, separately in each sex.” Although separate analyses do not allow for statistical comparison between females and males and in fact constitute a widespread and well-described logical error [10, 11, 20, 26–28], CIHR and LIBRA clearly considered such an approach an acceptable method to look for sex differences. CIHR stated, “Sex considerations [can] be taken into account by performing analyses in males and females separately;” in CIHR’s quizzes, approaches with separate analyses were marked as “correct,” e.g., a proper sex comparison can be achieved by “separating the data into two groups and then running the analyses separately for each group.” These quizzes required the acknowledgment of such analytical approaches as a “strength.” According to LIBRA, “disaggregating the data by considering the sexes separately can unmask sex differences.” In a LIBRA quiz, learners were asked to draw conclusions about sex differences from separate analyses of males and females. Notably, Module 3 of LIBRA consisted of two case studies presented as examples of how sex can and should be considered in research; both studies made claims that the sexes differed when the sexes were not quantitatively compared.

Despite the emphasis on separate analyses of data from females and males, all three trainings also covered a more appropriate approach: factorial designs that included sex-by-treatment interactions. Such approaches were typically presented as alternatives to separate analyses that should be used only under certain conditions, however. NIH, for example, mentioned testing for an interaction only after the sexes were analyzed separately. Similarly, LIBRA advised learners to first study outcome measures separately in each sex, then “compare, via statistics, outcome measures in females and males to establish the presence of sex differences.” Both NIH and LIBRA endorsed factorial designs only for studies *powered* to detect significant sex by treatment interactions; NIH suggested comparing outcome measures between sexes “using statistical tests” only in the context of a powered, confirmatory study. One statement in Module 3 stood out, however: “It may not be possible to power your study to detect a meaningful interaction between sex and treatment. In these cases, you should add a sex-by-treatment interaction term in the statistical model.” Thus, NIH seemed to argue here that interaction terms should be included only when underpowered, conflicting with other directives in the training. In a quiz, NIH recommended adding a sex-by-treatment interaction term in the statistical model “in all studies that consider SABV,” which appeared to conflict with other slides stating that interaction terms be included only under certain conditions. CIHR noted similarly that although comparing the sexes statistically should, theoretically, always be done, testing for interactions requires a larger sample size than conducting analyses within sex (which is not accurate, if the goal of within-sex analyses is to detect either an effect of a manipulation or a sex difference in that effect). CIHR stated further that interaction terms were generally not preferred because they are less “intuitive” and more “difficult to calculate and interpret” than the results of separate analyses. “When analyses are presented separately by sex,” CIHR explained, “this provides the clearest picture of where exposures might differ for men and women.”

In addition to strongly recommending that all data be *analyzed* separately for males and females, all three trainings emphasized the importance of separate *reporting*. All trainings recommended publishing raw data with the sex indicated for each sample as well as disaggregated demographic and descriptive data, which facilitates meta-analysis. But all three trainings went well beyond that minimum to recommend that all results be presented by sex as well, for example in separate graphs. The Primer even emphasized that reporting results “by sex” is a minimum requirement for compliance with SABV policy. Only the Primer mentioned being cautious about separate reporting, suggesting on one slide that sex-based analyses should be provided “as supplemental information, along with appropriate caveats, [which] allows you to share information that may inspire new hypotheses without overreaching your original study design.”

#### Reporting the sex of research participants and nonhuman animals

Each training mentioned the importance of reporting the sex of samples/participants. CIHR advised, “Always report the sex of animals, tissues, or cells used in the study.” LIBRA similarly stated, “Always report the sex of the cells, tissues, and animals you used, as well as the gender of human participants.” LIBRA also encouraged proper reporting, calling the practice of omitting sex information “sex insensitivity.” The NIH Primer noted that reporting sex is a requirement of some journals. Both NIH and LIBRA referred to the Sex and Gender Equity in Research (SAGER) guidelines, pointing out that they recommend sex be reported in the title of publications. NIH recommended that for single-sex studies, the word “specific” be included in the title, e.g., “Male-specific deficits in reward learning in a mouse module of alcohol use disorder,” even though a single-sex study cannot demonstrate that any finding is specific to one sex.

There was comparatively little coverage of reporting *how* sex was determined. The NIH Primer briefly advised researchers to “report operational definitions” of sex, emphasizing the contexts of cell culture and human studies in particular. LIBRA mentioned reporting operational definitions only in passing, in a single sentence inside a lecture. CIHR did not cover reporting definitions other than to recommend that researchers “properly identify” the sex of the animals or tissue/cell donor.

#### Reporting negative findings

All three trainings emphasized that the results of sex comparisons should be reported even when null. CIHR stated, “Report what you find, including null findings related to sex differences,” and “Any sex differences or similarities found, including null findings, [should] be reported in resultant publications to reduce publication bias, enable meta-analysis, support the identification of confounding variables and advance understanding.” The NIH Primer admonished, “Remember, analyses that do not indicate the presence of a sex difference are just as important as those that identify a sex difference… Report not only when you have identified a possible difference, but when your analyses suggest no difference according to sex.” The Primer went on to say, “To avoid needless repetition of studies by other investigators, report when analyses indicate the presence of a sex difference and when analyses suggest no difference based on sex.” LIBRA similarly stated, “Sex- and gender-based analyses should be reported regardless of positive or negative outcome.” Although there was no explicit instruction about the statistical invalidity of accepting a null hypothesis, we noted caveats about assuming that a null result shows good evidence for a sex similarity. For example, LIBRA warned that “if you happen to observe no sex differences this does not mean that they are not present in the process under investigation.” NIH also stated that “A lack of information supporting a sex-based difference in a biological process is not evidence that no difference exists,” and instructed researchers to “consider reviewing epidemiological work in the subject area for any evidence of sex-skewed incidence, prevalence, or outcomes.”

#### Discussion of limitations

As noted above, all three trainings endorsed exploratory subgroup analysis, even when underpowered, to test for potential sex differences. Only NIH mentioned the limitations of this approach. The Primer noted that authors must report whether sex differences were hypothesized *a priori* and whether the study was powered to detect sex differences, as well as explain that post-hoc findings are exploratory until replicated. NIH further stated that authors must “interpret and report findings within the specific limitations of the study’s design,” and “discuss appropriate generalizations as well as limitations.” Importantly, NIH recommended discussing “the potential influence of variables that interact with or are impacted by sex in your results.” Nonetheless, despite these caveats, there was a pervasive lack of specificity. No particular limitations were discussed, including those relevant to subset analysis – only that “subset analysis should be reported with appropriate restrictions.” Examples were not provided.

#### Talking to the media

The NIH Primer was the only training that referenced talking to the media. It noted that specific guidance about speaking to the media is lacking. It advised that when researchers do share their work with the public, they should be conservative and not go beyond what the data show.

### Potential pedagogical issues

#### Uneven levels of expertise assumed

Although the majority of each training seemed to assume little experience with study design or handling of data, both the CIHR and NIH courses sometimes seemed uneven in their assumptions about audience. For example, although it did not seem to assume any statistical expertise elsewhere in the course, the Primer contained the following unexplained terms on a single slide in Module 3: “superiority,” “non-inferiority,” “equivalence of treatments,” “one-sided” vs. “two-sided t-tests,” “minimum clinically important effects,” “repeated measures,” “clusters,” and “correlations between measurements.” These terms were included in a complex figure on how to calculate power to detect a main effect of treatment; the figure did not address power to detect sex differences. A button linking to the “3R’s” for more information was not functional, leading to an HTTP 503 error. On a single slide in Module 4, which covered reporting, we noted the unexplained terms “biological replicate,” “pseudoreplication,” and “interim analysis,” again outside the context of SABV specifically. Although each of these terms might be accessible to certain subgroups of scientists, the level of the vocabulary was uneven with respect to the rest of the Primer, which took an elementary approach overall. In addition, although the SABV policy (and presumably the training) was intended to reach preclinical researchers, there were many references in the Primer to terms such as “drop-out rates,” “stratification” and “confidence intervals,” which are typically used in clinical research. CIHR and LIBRA were more internally consistent in the level of knowledge they assumed, although CIHR used some undefined statistical terms such as “multivariate regression analysis” and “second-level sub-group disaggregation.”

#### Presenting material only in quizzes

A large percentage of the CIHR material consisted of quizzes. In most cases, the questions asked in the quizzes had not been covered by the preceding material; that is, the material was presented only in the context of a quiz. In those cases, CIHR seemed to expect learners to master the material by trial and error.

#### Interpretation of example data

**Fig. 1:**
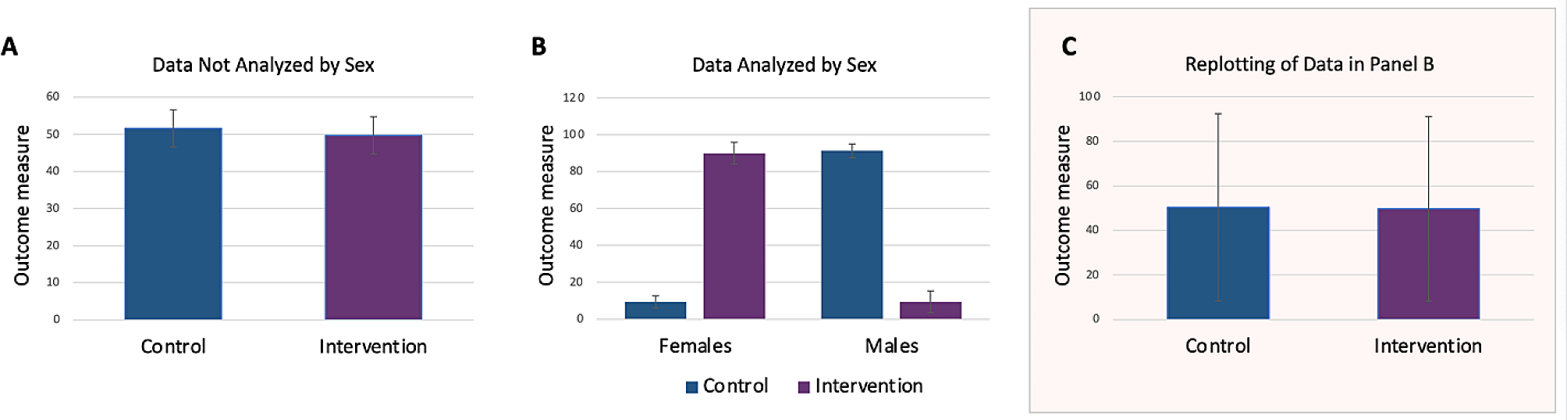
An impossible dataset. The NIH Primer illustrates the idea that when data from males and females are pooled, sex differences can be masked. (**A**) depicts a graph featured in a slide from the Primer Module 3 (see Fig. S1A). On this slide, which is part of a quiz, learners are asked to draw a conclusion about whether the intervention had an effect. No statistical results are presented. The “correct” answer is that the intervention had no effect. In the explanation of the correct answer, learners are told that such a result would be “evidence for the null hypothesis.” (**B**) depicts a graph from the next quiz question (see Fig. S1B), which claims to contain “the data from the same experiment disaggregated by sex.” Learners are again asked to draw a conclusion without seeing the results of statistical tests. The “correct” answer is that the intervention had an effect. Our analysis of the dataset presented in (**B**) (see Supplemental Methods) shows that the sex difference in the control group in (**B**) would be one of the largest quantitative sex differences ever described in any species (Cohen’s *d* = 23.24). After the intervention in (**B**), the sex difference flips to what would again be one of the largest ever measured, but in the opposite direction. (**C**) We reconstructed the dataset shown in (**B**) (see Fig. 1 Supplemental Methods and Table S3) and plotted the data pooled by sex with accurate error bars, showing the impossibility that the dataset in (**A**) could be the same as in (**B**)

NIH and LIBRA repeatedly asked learners to draw conclusions about a study’s findings on the basis of bar graphs alone, without the statistical results that should accompany the presentation of the data. In one of the LIBRA case studies, for example, conclusions were drawn without sufficient evidence: although no quantitative sex comparisons were presented, the commentary noted that “global demethylation is more pronounced in female cells.” Similarly, in the NIH Primer, example graphs were presented without the *F* or *p* values necessary to interpret the result. The NIH graphs sometimes suggested outcomes different from those stated in the commentary, e.g., whether effects were significant. In one particularly interesting example, the “same” data were graphed before and after disaggregating by sex. However, it was obvious that the two datasets could not be the same (Fig. 1).

There was some confusion, particularly in the NIH Primer, regarding the interpretation of ANOVA results. For example, Module 3 Lesson 4 of the Primer presented an example in which “the results of the two-way ANOVA indicated only the main effect of sex, no main effect of drug, and no interaction… In this case, females have a different response to the drug than males.” Absent a statistically significant sex-by-drug interaction, however, there would be no evidence that the females and males responded to the drug differently. A main effect of sex tells us that the outcome measure differed between sexes independently of the treatment, not that the difference was related to treatment (i.e., it was likely pre-existing).

Similar mix-ups between main effects, interactions, and post-hoc comparisons of means permeated the NIH Primer. For example, the Primer stated that comparisons of the outcome measure itself between males and females will indicate whether there is a “sex difference in the treatment” (presumably the *response* to treatment was intended). Further, as noted above, the training emphasized the importance of statistical power to detect sex by treatment interactions but the instructions on calculating power pertained only to the detection of main effects. The following text appeared near the end of the section on factorial designs (parenthetic statements added): “The ability of [factorial] analysis to determine the extent to which the outcome is altered by being male or female (main effect of sex) AND receiving drug or no drug (main effect of treatment) is invaluable to researchers examining the influence of sex on a potential treatment.” Note that “the influence of sex on a potential treatment” refers to the interaction, not the main effects; main effects do not give relevant information about sex-specific responses.

#### Endorsement of flawed research designs and interpretations

We noted multiple endorsements of flawed experimental design and interpretation of results, particularly in the NIH Primer. One of the quizzes in the Primer, for example, asked learners to identify the best design for specifying the most effective dosage of a drug. The “correct” answer was a study with only two drug conditions: no drug (control) and drug (treatment). To find the most effective dose, however, more than one dose must be tested. Another part of the NIH training recommended pooling samples of the same sex together (i.e., in cell culture), which violates assumptions of statistical independence across sources of tissue and confounds sex with other variables, such as culture plate, making it difficult to isolate sex as a variable of interest. Finally, in Module 3, the Primer stated that “a statistical comparison would indicate evidence for the null hypothesis,” indicating a problematic interpretation of the assumptions and methodology underlying null hypothesis significance testing.

### Other online courses

Our internet searches produced hits related not only to online training materials but also to other resources such as peer-reviewed literature reviews, opinion pieces, and government reports. Table S4 summarizes the most notable resources.

## Discussion

In this study, we have evaluated three sets of training materials intended to facilitate implementation of sex-based research policies in Canada, the United States, and the European Union. We found that overall, the courses were remarkably similar, focusing largely on the historical justification for the policies and the benefits of including females and males in research. All three trainings emphasized the potential harms of single-sex research, the implications for rigor and reproducibility, and the potential benefits of developing treatments tailored to a particular sex. The trainings were generally lacking, however, with respect to clarity and accuracy of recommendations for implementation of the policies, particularly regarding practical matters such as operationalization of sex, research design, and analytical approaches.

As CIHR, NIH, and other funding agencies revise and refine online training materials, opportunities will arise to incorporate additional perspectives into the course offerings so that the trainings evolve along with the needs of researchers. The time is also ripe for other entities, beyond funding agencies, to develop resources that fill current gaps. Here, we outline four areas that have recently increased tremendously in their visibility and in which researchers have expressed clear interest [16–20, 29, 30]: (1) The precise and contextualized operationalization of sex; (2) The risks inherent in a “two sizes fit all” approach to sex-based research and clinical applications; (3) The entanglement of sex and gender; and (4) Attention to rigorous analytical approaches to sex-based data. We outline each of these below, in hopes that future course offerings, both new and revised, can incorporate these important topics.

*Operationalization of sex*. None of the three trainings evaluated here devoted appreciable space to the operationalization of sex – either the importance of doing so or how it should be done. Given that the topic is not only controversial but often misunderstood, high-quality training in this area is urgently needed. The practice of defining and operationalizing sex in a research context has recently received considerable attention from a variety of perspectives [16–19]. Most of these authors emphasize a strategy in which “sex” is operationalized not by a crude category, which conflates several variables, but by a precisely defined sex-related variable such as the presence or absence of a Y chromosome or plasma levels of a hormone. This approach does not deny the multidimensionality of sex, require sex to be defined as non-binary, or impose the same operationalization on every researcher; on the contrary, it simply demands precision and accountability both in the collection and the reporting of data. Any updated trainings on how to incorporate sex as a variable in research will need to cover this topic, as the precise operationalization of all variables has a direct impact on rigor and reproducibility for any study.

*Potential downsides of sex reification*. The trainings we evaluated all emphasized the benefits of treating sex as the most important source of variation among people, other organisms, and cells. The primacy afforded to sex was apparent in statements that findings of single-sex studies are incomplete, erroneous, and even meaningless. Particularly salient were the admonitions that single-sex studies in non-human animals are not relevant to humans of all sexes; each training seemed to endorse the view that all physiological mechanisms interact with sex-related factors in identical ways in all species—that is, that being “male” or being “female” are essential qualities of an organism that generalize across taxonomic groups. All three trainings portrayed females and males as fundamentally and obviously “different.” Such characterizations of sex categories, without consideration of context and overlap, over-emphasize difference such that the sexes are ultimately believed to be more different than they actually are [31] and encourage scientists to approach research with the assumption that men and women need sex-specific treatments in every respect. But overhasty sex-specific regimens risk inappropriate treatment of individuals who do not match the average for their sex category [19]. For example, halving the dosage of the sleep aid drug zolpidem for women may have led to underdosing of some women, putting them at risk for accidents related to undertreated insomnia [32]. Consideration of such risks, particularly the risks of generalizing sex differences from non-human models to other species, should be incorporated into updated trainings.

*Entanglement of sex and gender*. While all three trainings noted that sex and gender are intertwined in complex ways, none explored the concept with appreciable depth. Instead, there was much space devoted to the *distinction* between sex and gender, with the former defined as anything “biological.” This definition was taken to extremes in that anything relating to the body, such as kidney function or viral load, was deemed “sex” despite being sensitive to gendered exposures. Examples of gendered exposures manifesting as sex differences were presented, but only as rare exceptions. Moving forward, it may be beneficial to focus less on the task of disentangling sex and gender, which recapitulates an unfruitful nature/nurture conundrum, and more on ways in which gender/sex as a single entity can be considered as complex, multidimensional, and socially embedded [14, 15].

*Rigor in analytical approaches*. All three trainings we evaluated for this study argued unambiguously and repeatedly that, regardless of statistical power, results should always be disaggregated by sex and presented separately. The trainings either explicitly or implicitly suggested that separate, within-sex analyses are an appropriate method to “reveal” sex differences. This analytical approach, which has been referred to as the “Difference in Sex-Specific Significance” (DISS) error [19, 20, 26, 33] is a version of one of the most common statistical errors in biomedical research [27]. Briefly, it involves separate tests of an effect of treatment or an exposure, followed by a qualitative comparison of *p* values between the sexes (significant or not); the sexes are not compared statistically at all. Several recent studies suggest that this error is made in approximately 70% of SGBA/SABV-compliant studies with factorial designs [8, 10, 11]. Because the DISS error markedly increases the risk of a false positive finding of “difference” [10, 28], there is a real risk that a large percentage of reported sex differences are not replicable, creating a “literature of contradiction” [34]. Given that enhancing rigor and reproducibility is a stated goal of sex and gender inclusion policies [1, 2, 4], this issue would seem to merit more coverage in the trainings. Currently, the online training courses we evaluated are much more concerned about false negatives, that is, missing a sex difference that is actually there, than false positives, that is, reporting a spurious one. But for the reasons noted in the section above, reporting false positive findings risks amplifying small or non-existent differences between women and men, perpetuating misguided notions of difference and potentially leading to unwarranted restrictions on which treatments are available to which patients [35]. Training materials, particularly those associated with government funding agencies, could attempt to mitigate this risk by providing instructions on how to consider sex appropriately in research designs and interpret results.

A main goal of the trainings we evaluated was to improve uptake – that is, to increase the extent to which females are included in research. These efforts are paying off; women are now equally represented in clinical trials [36] and female non-human animals are increasingly being included in preclinical research [9]. Thus, there is now an opportunity to help ensure that sex-based research policies attain their *other* intended goals, namely to increase rigor and reproducibility of biomedical research and to reduce harm to women and other marginalized groups. As the transdisciplinary field of sex differences research evolves, conceptualization of both sex and gender are rapidly changing [16–19, 29–31, 33]. The shortcomings that we have identified in these trainings may be due in part to the fact that the materials were developed at different stages of understanding of sex and gender in research and by a wide range of contributors, each with their own level of knowledge and area of expertise. The trainings would therefore benefit from revisions that reflect updated knowledge and awareness of key issues, particularly with respect to consideration of relevant variables beyond sex and the importance of gender-related variation. Beyond revising each of the trainings, it may be most impactful for the wording of the policies themselves to be clarified to ensure that they consistently reflect new understandings and consensus. For example, while gender has always been at the forefront of CIHR’s policy, the SGBA framework was updated to become “SGBA Plus” in 2021 [37] to emphasize intersectionality (with the “Plus” referring to additional intersecting factors such as age, race, ethnicity, disability, etc.). For its part, ORWH has recently begun to devote substantial attention and resources to topics such as the consideration of gender in research [38–40] and the impacts of structural sexism and relational power dynamics on women’s health [41, 42]. Formalizing these priorities, not only in the online training materials but in the policies themselves, would be an effective and productive way to provide scientists with clear guidance on how to thoughtfully and rigorously consider sex and gender in their research.

## Perspectives and significance

Recent implementation of sex-based research policies by a variety of government funding entities has created a need for researchers to be trained in the appropriate methodologies. Here, we have evaluated training materials provided directly from two federal bodies, CIHR and NIH, as well as materials made possible by funding from a third government entity, the European Commission. We found that all three sets of training materials emphasized the same rationales for the policies and relied on largely the same arguments and examples, indicating consensus. None of the trainings adequately covered the downsides or risks of focusing on sex category as the most important variable in every study. We identified other gaps in the coverage of sex-based research that should be filled, including how to precisely operationalize sex in the context of a research study and how to test for potential differences between sex or gender-classified groups using rigorous analytical approaches. Particularly regarding the latter, we noted that current training materials endorse methodologies that reduce, instead of enhance, rigor and reproducibility. Therefore, we call for revision of these materials, as well as the development of new ones, that attend more closely to these topics.

### Electronic supplementary material

Below is the link to the electronic supplementary material.

Supplemental File 1: Summaries of reviewed online trainings. This file contains detailed descriptions of each of the trainings we evaluated, along with links to each

Table S1: Codes used during evaluation of the three training courses

Table S2: Search terms used to search for relevant materials online

Table S3: Dataset generated for Fig. 1

Table S4: Selected highlights of online resources identified through literature searches

Figure S1 and Fig. 1 Supplemental Methods: Screenshots of the pages of the NIH SABV Primer on which Fig. 1 is based. This file also contains Supplemental Methods for Fig. 1

## Data Availability

The materials analyzed for the current study are publicly available at the following links: NIH: https://orwh.od.nih.gov/e-learning/sex-as-biological-variable-primer. CIHR: https://cihr-irsc.gc.ca/e/49347.html. LIBRA: https://www.libra-sgr.eu/libra/.
