# Supplementary material for: Training in the implementation of sex and gender research policies: an evaluation of publicly available online courses
Source: Biol Sex Differ. 2024 Apr 3;15:32. doi: 10.1186/s13293-024-00610-6 (PMC10988906; doi:10.1186/s13293-024-00610-6)
Supplement: Supplementary file 1 — Supplemental File 1: Summaries of reviewed online trainings. This file contains detailed descriptions of each of the trainings we evaluated, along with links to each [file 13293_2024_610_MOESM1_ESM.pdf]

SUPPLEMENTAL FILE 1: Summaries of reviewed online trainings

CIHR

https://cihr-irsc.gc.ca/e/49347.html

Integrating Sex & Gender in Health Research is an online training developed by the Canadian Institutes of Health Research (CIHR) to educate researchers and peer reviewers on how to account for and assess sex and gender across health research. The training can be found on the CIHR website under "learning," which can be found under the "funding" tab. A free account is required to access the training.

The training consists of three courses that participants click through at their own pace. The courses are set up as slideshow presentations with text, images, and narration. Each course also includes several multiple choice and short fill-in questions throughout. A multiple-choice post-test is located at the end of each course with feedback after all questions have been answered. Participants can take the test as many times as needed.

Course 1: Sex and Gender in Biomedical Research is intended for those conducting basic science research with cells, tissues, animals, and/or other biological samples. Course 1, which consists of an introduction, a pre-test, three objectives with educational content, a conclusion, and a post-test, takes approximately 45 minutes to complete. Objective 1: Recognize Nomenclature Used in Sex and Gender Science consists of 13 slides covering the definitions of sex and gender and takes approximately six minutes to complete; Objective 2: Identify Methods to Conduct Sex and Gender Science consists of 20 slides covering how to integrate sex and gender in various steps of research and takes approximately six minutes to complete; and Objective 3: Critically Appraise the Integration of Sex and Gender in Protocols and Publications consists of 28 slides covering sex and gender considerations in research proposals and takes approximately 15 minutes to complete. The post-test includes 11 multiple choice questions.

Course 2: Sex and Gender in Primary Data Collection with Human Participants is intended for those conducting clinical research collecting primary data from human participants. Course 2, which consists of an introduction, pre-test, three objectives with educational content, a conclusion, and a post-test, takes approximately 30 minutes to complete. Objective 1: Define and Distinguish Between Sex and Gender-related Variables in a Health Research Context consists of nine slides covering the differences between sex and gender and gender norms and roles, and takes approximately five minutes to complete; Objective 2: Identify Methods for Integrating Sex and Gender in Research Involving Primary Data Collection with Human Participants consists of 19 slides covering how to include and report sex and gender throughout human research and takes approximately 10 minutes to complete; and Objective 3: Critically Appraise the Integration of Sex and Gender in Protocols and Publications consists of 10 slides covering sex and gender considerations in research proposals and takes approximately four minutes to complete. The post-test includes 11 multiple choice questions.

Course 3: Sex and Gender in the Analysis of Secondary Data from Human Participants is intended for those conducting secondary data analyses of existing data sets with human participants. Course 3, which consists of an introduction, pre-test, three objectives with educational content, a conclusion, and a post-test, takes approximately 45 minutes to complete. Objective 1: Define and Distinguish Between Sex-related and Gender-related Variables Using Data from Human Participants consists of nine slides covering the use of sex and gender-related variables as units of analysis and takes approximately six

minutes to complete; Objective 2: Apply Methods for Conducting a Sex and Gender-based Analysis Using Data from Human Participants consists of 19 slides covering different methods to analyze sex differences and takes approximately 12 minutes to complete; and Objective 3: Critically Appraise the Integration of Sex and Gender in the Analysis Plan of Research Using Data from Human Participants consists of 10 slides covering sex and gender considerations in research proposals and takes approximately four minutes to complete. The post-test includes 11 multiple choice questions.

Participants earn a certificate after each course for a total of three.

NIH Primer

https://orwh.od.nih.gov/career-development-education/e-learning/sabv-primer

Sex as a Biological Variable: A Primer is an online training developed by the National Institutes of Health (NIH) Office of Research on Women's Health (ORWH) to educate researchers, NIH grant applicants, peer reviewers, and other members of the biomedical community on how to better account for the NIH sex as a biological variable (SABV) policy. The training can be found on the NIH ORWH website under "E-learning" under "career development programs and projects." A free account is required to access the training.

The SABV Primer training consists of four modules that participants click through at their own pace. The modules are set up as slideshow presentations with text, images, and narration. Each module also includes several multiple-choice questions throughout to test participants' retention of the previously presented information. A multiple-choice quiz is located at the end of each module with instant feedback after each question. If a question is answered incorrectly, participants can immediately try again. References are listed on individual slides as needed.

Module 1: SABV and the Health of Women and Men consists of five lessons, with the first four lessons presenting educational content and the fifth lesson including the end of module quiz. Module 1 takes approximately 20 minutes to complete. Lesson 1: Sex and Gender in Biomedical Research consists of seven slides covering the definitions of sex and gender and examples of health conditions impacted by both sex and gender, and takes approximately six minutes to complete; Lesson 2: NIH Sex as a Biological Variable (SABV) Policy consists of four slides covering a brief introduction to the NIH SABV policy and takes approximately two minutes to complete; Lesson 3: SABV Policy: Concept and Policy Development consists of seven slides covering the history of the NIH SABV policy's development, reproducibility, and scientific rigor, and takes approximately seven minutes to complete; and Lesson 4: SABV Across the Biomedical Research Continuum consists of six slides covering the importance of including both sexes across the biomedical field and takes approximately two minutes to complete. Lesson 5: Review What You've Learned includes a quiz with four multiple choice questions.

Module 2: SABV and Experimental Design consists of six lessons with similar formatting to Module 1. Module 2 takes approximately 30 minutes to complete. Lesson 1: Consider and Collect Data on Sex for Strengthening Science consists of six slides covering how to consider sex in various research designs and collect data to allow for disaggregation by sex and takes approximately three minutes to complete; Lesson 2: SABV in Experimental Design consists of eight slides covering how to include both sexes in experimental designs such as factorial and adaptive designs and takes approximately five minutes to complete; Lesson 3: A Checklist to Enhance SABV in Study Design consists of 18 slides covering the steps to satisfactorily include SABV in a study design with examples and takes approximately nine minutes to complete; Lesson 4: Common Misconceptions About SABV consists of five slides covering misconceptions about the inclusion of SABV in study designs and takes approximately two minutes to complete; and Lesson 5: SABV Policy Across the Translational Science Spectrum consists of four slides

covering the implementation of SABV at all stages of biomedical research and takes approximately 10 minutes to complete. Lesson 6: Review What You've Learned includes a quiz with four multiple choice questions.

Module 3: SABV and Analyses consists of five lessons, with similar formatting to the earlier modules. In its entirety, Module 3 takes approximately 20 minutes to complete. Lesson 1: Explain the Rationale for Characterizing and Analyzing Sex-Based Data consists of two slides covering the interpretation of data by sex and takes approximately one minute to complete; Lesson 2: Understand the Limitations of Analyses that Ignore Sex Influences and Differences consists of five slides covering how failing to include SABV can result in erroneous conclusions and takes approximately three minutes to complete; Lesson 3: Utilize Statistical and Power Analyses to Detect Sex Differences consists of 15 slides covering power analyses, sample size, and analyses of variance and takes approximately nine minutes to complete; and Lesson 4: Apply Statistical Approaches to Measure Sex Influences and Differences consists of four slides covering studies designed to identify sex differences and takes approximately four minutes to complete. Lesson 5: Review What You've Learned includes a quiz with six multiple choice questions.

Module 4: SABV and Research Reporting consists of six lessons, with similar formatting to the earlier modules. In its entirety, Module 4 takes approximately 25 minutes to complete. Lesson 1: Communicate and Report Data by Sex consists of two slides covering rigor and reproducibility policies and takes approximately two minutes to complete; Lesson 2: Recognize how SABV Can Be Incorporated Into the Reporting of Results consists of three slides covering how to communicate sex-based data and takes approximately five minutes to complete; Lesson 3: Identify Basic Elements of Guidelines for Reporting Sex & Gender in Research consists of nine slides covering reporting and interpreting demographic data and takes approximately five minutes to complete; Lesson 4: Apply Guidelines for Publishing in Scientific Journals consists of 13 slides covering SAGER guidelines and journal standards and takes approximately 11 minutes to complete; and Lesson 5: Share Sex and Gender Information Outside of the Scientific Community consists of three slides covering how to discuss sex and gender with the media and takes approximately two minutes to complete. Lesson 6: Review What You've Learned includes a quiz with five multiple choice questions.

Participants must complete all four modules to earn a certificate.

LIBRA

https://www.libra-sgr.eu/libra/

The Sex and Gender Dimension in Biomedical Research is an online training developed as part of "Leading Innovative Measures to Reach Gender Balance in Research Activities" (LIBRA) from the European Commission." This course introduces users to basic concepts and examples of the Sex and Gender Dimension in Research (SGR), as well as how to account for sex and gender in experimental practice. The training can be found on the LIBRA website by clicking "LIBRA online module" under "LIBRA closing and resources." A free account is required to access the training.

The training consists of three modules that participants click through at their own pace. The modules consist of video lectures and discussions, which sometimes include a corresponding slideshow presentation. A multiple-choice test is located at the end of each module with feedback after all questions have been answered. Participants can take the test as many times as needed.

Module 1: SGR: The Context includes one lesson with educational content and a final test. Lesson 1 – Sex and Gender Dimension of Research: The Challenge consists of a video lecture by Sabine Oertelt-

Prigione, which lasts about 35 minutes and covers the distinctions between sex and gender, examples of SGR, and challenges of SGR. The final test includes five multiple choice questions.

Module 2: How to Integrate SGR Analysis in Research consists of three lessons with educational content and a final test. In its entirety, Module 2 takes approximately 40 minutes to complete. Lesson 1 – How to Integrate Sex Analysis in Research 1 consists of 30 slides covering common mistakes concerning SGR, SGR relevance, and SGR in literature, and takes approximately 13 minutes to complete; Lesson 2 – How to Integrate Sex Analysis in Research 2 consists of 37 slides covering accounting for sex in vivo and in vitro research and how to design statistical samples, and takes approximately 17 minutes to complete; and Lesson 3 – How to Integrate Sex Analysis in Research 3 consists of 25 slides covering how to report sex in research findings under SAGER guidelines and increasing SGR awareness, and takes approximately nine minutes to complete. The final test includes 10 multiple choice questions.

Module 3: Case Studies includes two case studies, one video lecture with educational content, and a final test. Case Study 1 – Sex Differences in Intestinal Adenomas includes 20 slides covering a scientific study conducted with sex and gender in mind to find sex differences in intestinal adenomas and takes approximately seven minutes to complete; Case Study 2 – Sex Differences in iPSC Reprogramming includes 23 slides covering another example of a study considering sex and gender to show sex differences in iPSC reprogramming and takes approximately 10 minutes to complete; and Expert Opinion – A Conversation with Gian-Paolo Dotto includes an interview with Gian-Paolo Dotto that lasts about 27 minutes and covers the importance of SGR and his project studying how genetic and epigenetic determinants of skin cancer can vary across gender and race. The final test includes eight multiple choice questions.

Participants must complete all three modules to earn a certificate.
