# Supplementary material for: Training in the implementation of sex and gender research policies: an evaluation of publicly available online courses
Source: Biol Sex Differ. 2024 Apr 3;15:32. doi: 10.1186/s13293-024-00610-6 (PMC10988906; doi:10.1186/s13293-024-00610-6)
Supplement: Supplementary file 2 — Table S1: Codes used during evaluation of the three training courses [file 13293_2024_610_MOESM2_ESM.pdf]

Gompers, et al.

Table S1: Codes used during evaluation of the three training courses.

code #	code name	description of code
0	**POLICY**	states what the policy is/what it covers and does not cover
0.1	other policies	discussion of policies other than the policy under question in the training, e.g., SAGER
1	**IMPORTANCE OF POLICY**	importance of/justification for considering sex as a variable in research
1.1	pitfalls	acknowledges potential pitfalls or cautions to keep in mind when applying policy
1.2	controversy	acknowledges controversy re: sex differences
1.3	examples	examples of sex differences that illustrate need to consider sex as a variable in research
1.31	drug sensitivities	references sex differences in clearance rate, adverse effects, etc. of any non-zolpidem drug
1.311	Zolpidem	mentions Ambien/zolpidem
1.33	AD	references sex differences in Alzheimer's Disease
1.34	pain	references sex differences in pain mechanisms, sensitivity, etc. (some overlap with drug code, e.g. opiates)
1.35	CVD	references sex differences in cardiovascular disease
1.36	mental health	references sex differences in mental health conditions, e.g., depression
1.4	bias in research	references historic or current failure to include women/females in research studies
1.41	neglect of women	notes sex bias in past or present clinical trials
1.42	neglect of female animals	notes sex bias in past or present preclinical research
1.5	return on investment	argues that failing to consider sex wastes time and resources
1.6	education	argues that sex differences need to be taught to doctors/med students
1.7	rigor	uses "follow the science" or "rigor" etc. to justify policy
1.71	generalizability of findings	argues that failing to stratify by sex or using just one sex makes the findings or conclusions questionable, erroneous, or less generalizable
1.8	precision medicine	uses SABV/sex as a broad category in support of personalized/precision medicine; sex-specific treatments. "Translational"
1.9	social justice	mentions social justice/inclusivity
1.91	vague risk of harm	mentions risk of harm that comes from not incorporating SABV without specific examples or further explanation
2	**SEX/GENDER**	conceptualization and operationalization of sex/gender; key terms
2.1	binarization	binary categories of sex or gender
2.11	gender spectrum	mentions "spectrum" of sex or gender
2.12	transgender	reference to trans people/populations; gender diversity
2.13	LGBQ	reference to diversity of sexualities
2.2	entanglement	acknowledges difficulty separating sex and gender
2.21	sex/gender interactions	acknowledges that sex and gender can interact and jointly influence health and biology
2.22	gender effects	acknowledges that gender can affect health independently of sex
2.3	operationalization	operationalizes sex and gender (defines terms)
2.31	oper. guidance	instructs learners to clearly operationalize sex/gender in their own work
2.32	sex: definitions	defines sex using a particular trait, e.g. chromosome complement vs. multidimensional
2.33	biologization of sex	defines sex=nature (biology); gender = nurture (culture); sex=biology; "biological sex"
2.34	sex slippage	the training itself confuses definitions of sex, e.g. slipping back and forth between hormones and chromosomes
2.35	other terms	e.g., explains when to use "men" vs. "males; "women" vs. "females"; or fails to explain/uses the terms interchangeably
2.36	gender: definitions	definitions of gender other than those that simply distinguish between sex and gender.
2.4	non-human gender	states a position about whether gender must be used exclusively for humans

3	**RESEARCH DESIGN & ANALYSIS**	guidance on study design and statistical analysis not otherwise coded below
3.1	single-sex studies	when they are appropriate (e.g., sex-specific ailments); when they are not (e.g., costs)
3.11	sex-specific conditions	examples of conditions that could justify a single-sex study
3.12	recognition of challenges	examples/permission to not account for sex, e.g. when it is too expensive/time-consuming to genotype cells, etc.
3.2	exploratory studies	distinguishes between exploratory studies (not intended to detect sex differences) and confirmatory (studies that are)
3.21	a priori hypotheses	mentions a priori hypotheses about sex and proposed mechanisms, etc.
3.22	literature search	design/hypotheses
3.3	"analyze data by sex"	reference to considering sex in data analysis with no further guidance about what that means in practice
3.31	disaggregated data (meta-analysis)	mentions publishing disaggregated data to enable future meta-analysis
3.4	Designs	e.g., explains factorial designs or other ways that sex is "accounted for"
3.41	sex x treatment interactions	specifically mentions testing for statistical interactions between sex and another variable of interest
3.42	sex as covariate	discusses including sex as a covariate or other method of "controlling for sex"
3.43	DISS	explicitly directs readers to analyze data within-sex, without mentioning testing first for statistical interactions
3.44	other statistical guidance	other directives, e.g. get confidence intervals, etc.
3.5	example datasets/graphs	visual depictions of example data
3.6	power	mentions of power, power analyses
3.7	non-sex variables	acknowledges variables other than sex that could affect outcomes; e.g. gendered professions, smoking, body weight
3.71	non-sex variables - guidance	provides guidance on how to account for other variables while also accounting for sex
3.8	estrous cycles	mentions need (or lack of need) to track estrous cycles in preclinical research
4	**REPORTING & INTERPRETING DATA**	guidance on reporting findings not otherwise coded below
4.1	exploratory findings	explains how to report findings from an exploratory study
4.11	exploratory-limitations	specifically explains what are the limitations of drawing conclusions from exploratory studies
4.12	DISS-presentation	advises readers to present within-sex analyses even for exploratory studies
4.2	interpretations	guidance on interpretating results
4.21	non-sex explanations	advises readers to consider gendered variables as potential explanations for results
4.22	mechanisms	advises to consider mechanism for sex differences
5	**MISC. CODES**	miscellaneous not coded above or below
5.1	reification	suggests that sex is the most important or only factor to consider in research
5.2	conflict	guidance/information conflicts with other guidance/information in the training
5.3	fundamental differences	argues that the sexes are "fundamentally" different or other wording to suggest very large sex differences
5.31	"every cell has a sex"	and similar arguments that the entire body is sexually differentiated
5.4	overstated claims	claims not supported by evidence
5.5	missing information	leaves out relevant important information
5.6	inaccurate information	information that is demonstrably, irrefutably wrong
5.7	sex similarity	acknowledges that the sexes are not always different on every measure
