# Supplementary material for: Training in the implementation of sex and gender research policies: an evaluation of publicly available online courses
Source: Biol Sex Differ. 2024 Apr 3;15:32. doi: 10.1186/s13293-024-00610-6 (PMC10988906; doi:10.1186/s13293-024-00610-6)
Supplement: Supplementary file 3 — Table S2: Search terms used to search for relevant materials online [file 13293_2024_610_MOESM3_ESM.pdf]

Gompers, et al.
Training in the implementation of sex and gender research policies: An evaluation of publicly available online courses

Table S2: Search terms

Search term	Date of search
Austria sex-gender dimension training	1/3/2023
Austrian Research Promotion Agency gender research	1/3/2023
Deutsche Forschungsgemeinschaft sex dimension training	1/3/2023
France sex-gender dimension training	1/3/2023
GBA plus science training	1/2/2023
Germany sex-gender dimension training	1/3/2023
Inclusion of sex in biomedical research best practices	12/6/2022
Integrating gender analysis into research Spain	1/3/2023
Ireland sex-gender dimension training	1/3/2023
MRC sex requirements	1/2/2023
MRC sex requirements training	1/3/2023
MRC sex training	1/3/2023
Norway sex-gender dimension training	1/3/2023
Research Council of Norway gender policy	1/3/2023
Research Council of Norway gender policy training	1/3/2023
SABV analysis	12/28/2022
SABV course	11/8/2022
SABV how to	12/1/2022
SABV implementation	12/1/2022
SABV modules	11/27/2022
SABV preclinical research training	10/24/2022
SABV quizzes	12/28/2022
SABV resources	12/1/2022
SABV training	10/17/2022
SABV training	10/24/2022
SAGER course	1/1/2023
SAGER modules	1/1/2023
SAGER research course	1/1/2023
SAGER research modules	1/1/2023
SAGER research trainings	1/1/2023
SAGER trainings	1/1/2023
SGBA science training	1/2/2023
SGBA training	1/2/2023
SGBA training Canada	1/3/2023

SGBA training for researchers	1/3/2023
SGBA training for researchers Canada	1/3/2023
Sex and gender based analysis training	1/3/2023
Sex and gender equity in research course	1/1/2023
Sex and gender equity in research modules	1/1/2023
Sex and gender equity in research training	1/1/2023
Sex as a biological variable analysis	1/1/2023
Sex as a biological variable course	12/28/2022
Sex as a biological variable how to	1/1/2023
Sex as a biological variable implementation	1/1/2023
Sex as a biological variable modules	12/28/2022
Sex as a biological variable preclinical research training	12/28/2022
Sex as a biological variable quizzes	1/1/2023
Sex as a biological variable resources	1/1/2023
Sex as a biological variable training	11/27/2022
Sex in research modules	1/1/2023
Sex in research training	1/1/2023
Sex in research training Canada	1/2/2023
Spain IGAR training	1/3/2023
Spain sex-gender dimension training	1/3/2023
