# Supplementary material for: Training in the implementation of sex and gender research policies: an evaluation of publicly available online courses
Source: Biol Sex Differ. 2024 Apr 3;15:32. doi: 10.1186/s13293-024-00610-6 (PMC10988906; doi:10.1186/s13293-024-00610-6)
Supplement: Supplementary file 4 — Table S3: Dataset generated for Fig. 1 [file 13293_2024_610_MOESM4_ESM.pdf]

Gompers, et al.

Table S3: Dataset generated for Fig. 1.

**sex**	**Tx**	**outcome**	**sex**	**Tx**	**outcome**
female	control	6.06	female	intervention	98.12
female	control	8.64	female	intervention	98.45
female	control	7.76	female	intervention	86.06
female	control	14.51	female	intervention	84.20
female	control	9.25	female	intervention	85.31
female	control	12.76	female	intervention	86.66
female	control	16.00	female	intervention	79.85
female	control	6.22	female	intervention	87.98
female	control	7.82	female	intervention	94.04
female	control	7.85	female	intervention	91.87
female	control	6.54	female	intervention	91.72
female	control	9.60	female	intervention	95.19
male	control	87.45	male	intervention	11.07
male	control	95.21	male	intervention	25.02
male	control	86.04	male	intervention	9.40
male	control	88.59	male	intervention	13.36
male	control	96.94	male	intervention	2.00
male	control	88.63	male	intervention	6.89
male	control	96.73	male	intervention	9.32
male	control	91.49	male	intervention	10.91
male	control	90.44	male	intervention	8.87
male	control	94.78	male	intervention	7.68
male	control	88.53	male	intervention	3.31
male	control	90.83	male	intervention	6.02
