# Supplementary material for: Training in the implementation of sex and gender research policies: an evaluation of publicly available online courses
Source: Biol Sex Differ. 2024 Apr 3;15:32. doi: 10.1186/s13293-024-00610-6 (PMC10988906; doi:10.1186/s13293-024-00610-6)
Supplement: Supplementary file 5 — Table S4: Selected highlights of online resources identified through literature searches [file 13293_2024_610_MOESM5_ESM.pdf]

Table S4
Selected highlights of online resources identified through literature searches. See Table S2 for search terms.

Organization	Description	Link
**Online courses**		
NIH ORWH	ORWH offers a "Bench to Bedside" series of courses, developed in conjunction with the Food and Drug Administration, that explores sex and gender differences in several disease areas.	bit.ly/4aaHLAi
NIH ORWH	ORWH also offers a newer course, "Train the Trainer," which is aimed not only at trainers but also researchers and grant applicants.	bit.ly/3uSV8VL
Global Preclinical Data Forum	Developed with R25 funding from NIH, this online course offers practical guidance to preclinical researchers on how to navigate the NIH's 2015 policy.	bit.ly/41cxdfX
Women's Xchange	With support Canadian Institutes of Health Research, the Women's Xchange, which is based at Women's College Hospital in Toronto, developed "The Health Researcher's Toolkit: Why Sex and Gender Matter" to provide researchers with strategies for collaborating with marginalized and vulnerable community members.	bit.ly/3GCdEUP
University of Dublin	Developed by Trinity College Dublin's Centre for Gender Equality and Leadership as part of the Systemic Action for Gender Equality (SAGE) Horizon 2020 project, "Creating a Gender Sensitive Institution" is an online course reviewing how to advance gender equality in a higher education workplace and research.	bit.ly/3uQ8d23
Other resources		
GENDER-NET	This EC-funded project offers tools and checklists for Integrating Gender Analysis into Research (IGAR).	bit.ly/3NmUyWC
Gendered Innovations	Stanford University started the Gendered Innovations project and has since collaborated with groups across the United States, European Union, Canada, and Asia to offer practical methods for sex and gender analysis in science and engineering.	stanford.io/3Rg0DFi
Specialized Centers of Research Excellence on Sex Differences (SCORE)	These NIH-funded centers offer a variety of resources for professional development. For example, the SCORE at the University of California, Los Angeles maintains a large video library consisting of basic, didactic information on research and clinical practice, as well as state-of-the-art lectures on the most recent research (https://bit.ly/4aaI3qS). The SCORE at Emory University hosts an annual virtual workshop on "How to Incorporate Sex as a Biological Variable (SABV) in Your Research" each December.	bit.ly/3GHC8vH

NIH ORWH: National Institutes of Health Office of Research on Women's Health
