# Supplementary material for: Training in the implementation of sex and gender research policies: an evaluation of publicly available online courses
Source: Biol Sex Differ. 2024 Apr 3;15:32. doi: 10.1186/s13293-024-00610-6 (PMC10988906; doi:10.1186/s13293-024-00610-6)
Supplement: Supplementary file 6 — Figure S1 and Fig. 1 Supplemental Methods: Screenshots of the pages of the NIH SABV Primer on which Fig. 1 is based. This file also contains Supplemental Methods for Fig. 1 [file 13293_2024_610_MOESM6_ESM.pdf]

Gompers, et al.
Training in the implementation of sex and gender research policies: An evaluation of publicly available online courses
Fig. S1 and Fig. 1 Supplemental Methods

A.

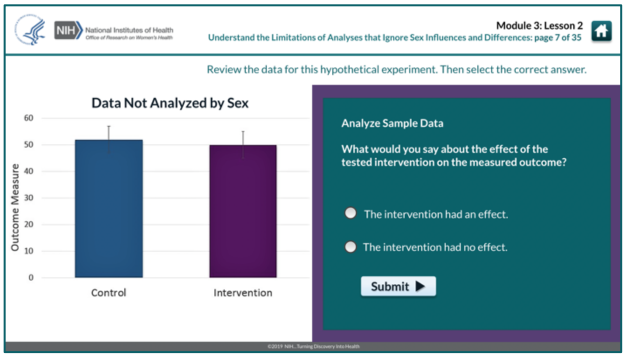

B.

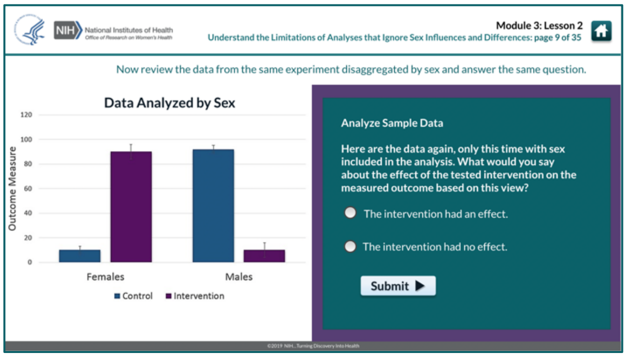

Fig. S1. Screenshots of the pages of the NIH SABV Primer on which Fig. 1 is based. The graphs in Fig. 1 in the main text are based on two figures from the NIH SABV Primer (Module 3, Lesson 2, pages 7 and 9; shown in A and B, above). We took screenshots of these figures and used ImageJ (NIH) to determine the mean of each group, as well as the sizes of the error bars. The "set scale" feature was used to calibrate the measurements using the Y axis in the images. We used these estimated values (Table S3) in Excel to correct the graph shown in 1A above (see Panels A and C of Fig. 1 and Supplemental Methods below).

Fig. 1 Supplemental Methods
To demonstrate that the two graphs in Fig. S1 cannot be drawn from the same dataset, we needed to generate sample data with the same means and variation as depicted in Fig. S1B. The Primer indicated neither sample sizes nor whether the error bars represent standard errors or standard deviations. Because the graphs in Fig. S1 are presumably based on Fig. 3 of McCullough et al. [1,2], we used that publication as a guide to choose a sample size of n = 12 and to depict standard deviation in the error bars. We used the Gaussian random numbers function at Random.org to generate four samples of data (female control, female intervention, male control, and male intervention) with means and standard deviations as similar as possible to the graph in Fig. S1B (Table S3). We then used those data to create the graph in Panel B of Fig. 1. Next, we pooled the sexes in that dataset and replotted the data; the resulting graph appears in the inset in Fig. 1.

References
1. McCullough LD, Zeng Z, Blizzard KK, Debchoudhury I, Hurn PD. Ischemic nitric oxide and poly (ADP-ribose) polymerase-1 in cerebral ischemia: male toxicity, female protection. J. Cereb. Blood Flow Metab. 2005; 25:502-12.
2. Clayton JA. Applying the new SABV (sex as a biological variable) policy to research and clinical care. Physiol Behav. 2018; 187:2-5.
